# Metabolic Consequences and Vulnerability to Diet-Induced Obesity in Male Mice under Chronic Social Stress

**DOI:** 10.1371/journal.pone.0004331

**Published:** 2009-01-30

**Authors:** Alessandro Bartolomucci, Aderville Cabassi, Paolo Govoni, Graziano Ceresini, Cheryl Cero, Daniela Berra, Harold Dadomo, Paolo Franceschini, Giacomo Dell'Omo, Stefano Parmigiani, Paola Palanza

**Affiliations:** 1 Department of Evolutionary and Functional Biology, University of Parma, Parma, Italy; 2 Department of Internal Medicine, Nephrology and Health Sciences, University of Parma, Parma, Italy; 3 Department of Experimental Medicine, University of Parma, Parma, Italy; 4 Department of Internal Medicine and Biomedical Sciences, University of Parma, Parma, Italy; 5 Ornis Italica, Rome, Italy; James Cook University, Australia

## Abstract

Social and psychological factors interact with genetic predisposition and dietary habit in determining obesity. However, relatively few pre-clinical studies address the role of psychosocial factors in metabolic disorders. Previous studies from our laboratory demonstrated in male mice: 1) opposite status-dependent effect on body weight gain under chronic psychosocial stress; 2) a reduction in body weight in individually housed (Ind) male mice. In the present study these observations were extended to provide a comprehensive characterization of the metabolic consequences of chronic psychosocial stress and individual housing in adult CD-1 male mice. Results confirmed that in mice fed standard diet, dominant (Dom) and Ind had a negative energy balance while subordinate (Sub) had a positive energy balance. Locomotor activity was depressed in Sub and enhanced in Dom. Hyperphagia emerged for Dom and Sub and hypophagia for Ind. Dom also showed a consistent decrease of visceral fat pads weight as well as increased norepinephrine concentration and smaller adipocytes diameter in the perigonadal fat pad. On the contrary, under high fat diet Sub and, surprisingly, Ind showed higher while Dom showed lower vulnerability to obesity associated with hyperphagia. In conclusion, we demonstrated that social status under chronic stress and individual housing deeply affect mice metabolic functions in different, sometime opposite, directions. Food intake, the hedonic response to palatable food as well as the locomotor activity and the sympathetic activation within the adipose fat pads all represent causal factors explaining the different metabolic alterations observed. Overall this study demonstrates that pre-clinical animal models offer a suitable tool for the investigation of the metabolic consequences of chronic stress exposure and associated psychopathologies.

## Introduction

The chronic activation of the stress response has been associated with metabolic disorders and altered energy homeostasis [1], [2]. Acute increase of stress hormones, such as glucocorticoids (GCs), catecholamines, etc. may determine the mobilization of fuel molecules, stimulate or inhibit feeding, and oppose insulin action [3]–[6]. However, sustained concentrations of GCs as observed under chronic stress can also increase the salience of pleasurable or compulsive activities (ingesting sucrose, fat, and drugs, or wheel-running). This, in synergy with insulin, may increase ingestion of “comfort food” and systemically increase abdominal fat depots [1], [6], [7]. Experimental studies in humans have demonstrated that perturbations of the hypothalamus-pituitary-adrencortical (HPA) axis function relate with abdominal obesity [8] and that stress perception strongly associates with a higher waist-to-hype-ratio and body mass index (BMI) [9], [10]. In addition, in patients depression has also been associated with the metabolic syndrome and obesity [1], with pre-existing differences in BMI predicting the direction of changes in energy balance determined by job stress [11]. Finally, in a cohort of Finnish twins discordant for adult BMI, the obese co-twins showed the highest index of psychosocial stress perception when compared to the lean co-twins [12].

Differently from humans, experimental models in animals offer the advantage to allow an easier manipulation of key experimental variables for the investigation of psychosocial factors affecting vulnerability to stress exposure [7], [13]–[16]. In particular, animal models of social stress appear to have a high validity as models of human psychopathologies [13]–[18]. Unfortunately, until recently there was a paucity of animal models in which stress exposure was associated with body weight gain. Indeed, animal models of chronic stress, including chronic subordination, have repeatedly been associated with a reduction in body weight and a generalized catabolic state [19]–[24]. This clear-cut effect is not present in the human literature and the DSM-IV defines weight gain or loss as a diagnostic criterion for major depression [25]. Recently, our and other laboratories described animal models for chronic stress-induced increase in body weight and adiposity [26]–[29] and vulnerability to diet induced obesity [28], [30], [31]. In addition, recent studies have showed neuroendocrine evidences of metabolic syndrome in defeated rats fed high fat diet but not a standard diet [32]. Furthermore, there is evidence that social status in models of chronic stress might differentially affect stress-induced metabolic effects: Bartolomucci et al [26], Moles et al [28] and Solomon et al [29] using similar experimental models in mice and hamsters, reported that subordination can be reliably associated with increased weight gain, whereas dominance is associated with lower weight gain or weight loss. However, there are currently no studies comparing different models of social stress that simultaneously determine behavioral, metabolic, biochemical and anatomical alterations in the experimental animals. Thus, the aims of the present study were: 1) to clarify the metabolic consequences of social stress using two models, i.e. chronic psychosocial stress distinguishing between dominants (Dom) and subordinates (Sub) [26], [33], and individual housing (Ind) [34]; 2) to characterize for the first time sympathetic system related parameters within visceral adipose fat pads in animals under chronic stress; 3) to determine morphological changes in the adipose tissue; and finally 4) to determine if the metabolic consequences of stress-exposure might translate into altered vulnerability to high fat diet (HFD)-induced obesity.

## Results

### Behavioral and endocrine consequences of chronic psychosocial stress

**Figure 1:**
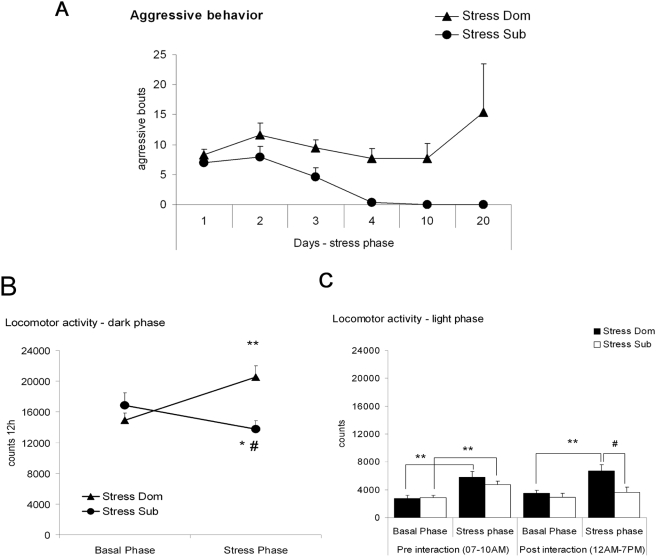
Behavioral consequences of chronic psychosocial social stress in mice. A) Aggressive behavior assessed on days 1 to 4, 10 and 20 of the stress phase. Graph clearly shows how dominants (Dom) and subordinates (Sub) are non-overlapping behavioral categories. B) Locomotor activity measured during baseline (4 days) and the stress phase (20 days). Dom showed increased and Sub showed decreased locomotor activity (F(1,18) = 21.9, p<0.01). C) Locomotor activity measured before and after the daily agonistic interaction. Dom showed increased activity both before and after the agonistic interaction while Sub showed increased activity before but not after the agonistic interaction (F(1,18) = 4.1, p = 0.054). * p<0.05 and ** p<0.001 vs. basal, # p<0.05 vs. Dom.

**Figure 2:**
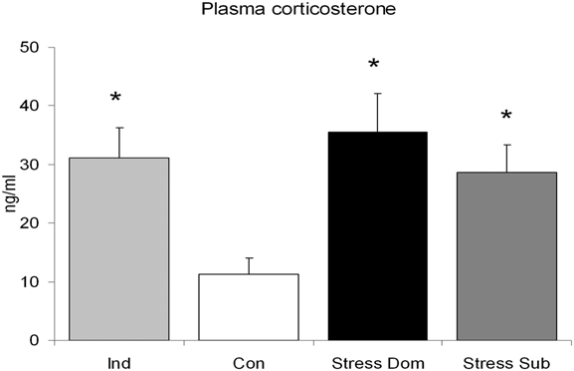
Hormonal consequences of social stress in mice. Basal plasma corticosterone collected in the early light phase, was increased in subordinates (Sub, U_9,13_ = 23, p<0.016), dominants (Dom, U_9,12_ = 12, p<0.016) and individually housed (Ind, U_9,5_ = 3, p<0.005) mice when compared to Controls (Con). * p<0.016.

According to our standard protocol [26], after a few days each dyad was clearly biased into a stable dominant/subordinate relationship, with Dom being the only mice showing aggressive behavior (Figure 1A). Individual locomotor activity was scored in the home cage by means of infrared sensors. The analysis revealed that in the dark phase (the active period for mice), Dom showed an increase in locomotor activity, while Sub showed a depression of locomotor activity when compared with baseline values (Figure 1B). A separate analysis of locomotor activity during the light phase revealed that Dom showed a strong stress-associated increase both before and after interaction. On the contrary, Sub showed increased activity only before, but not after, the daily fight which can be interpreted as an anticipation of the agonistic interaction [35] and imply a disturbance of the normal sleep pattern, i.e. reduced sleep during the early light phase (the normal inactive period for mice). In Sub the post-interaction light phase activity remained unaffected when compared with baseline but was clearly lower when compared with Dom (Figure 1C). Finally, both Dom and Sub showed increased basal corticosterone plasma level after 21 days of chronic stress exposure (Figure 2).

### Metabolic consequences of chronic psychosocial stress: social status effects

**Figure 3:**
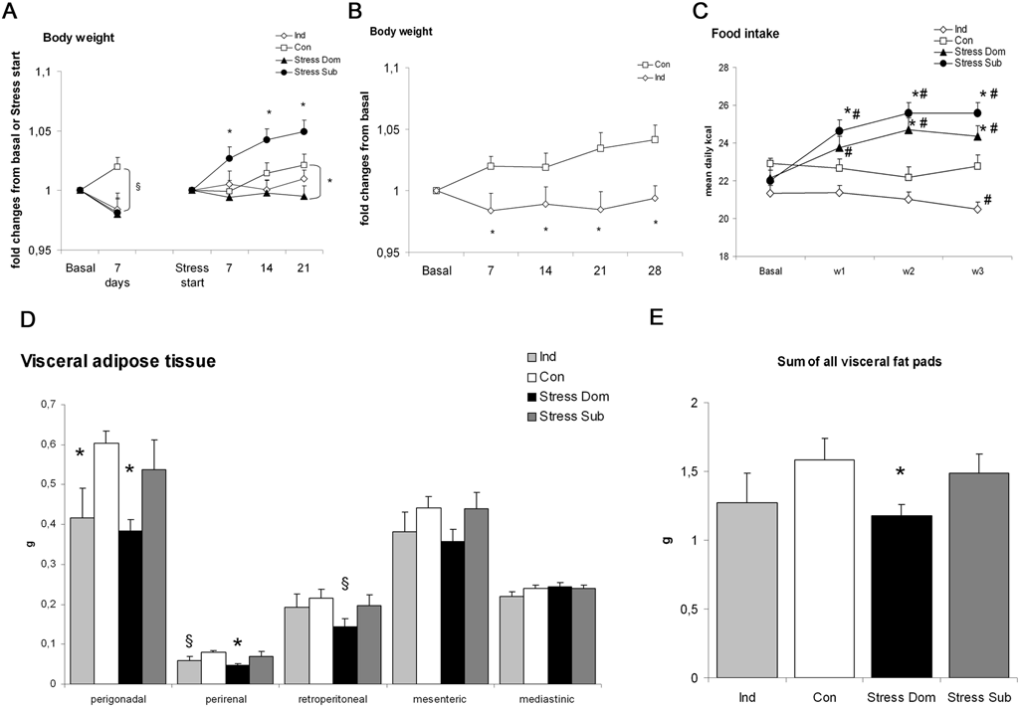
Metabolic consequences of social stress in mice. A) Body weight changes in the baseline and in the stress phase. At baseline, all experimental groups showed a trend for a lower body weight gain than controls (Con) (F(3,39) = 2.6, p = 0.06). In the stress phase, subordinates (Sub) showed a larger body weight gain when compared to all other groups, which were not different from each other (F(3,38) = 4.6, p<0.01). Figure describes only post hoc comparisons to controls, * p<0.05; § p = 0.06. B) Body weight changes from baseline in Con and individually housed (Ind) mice starting from the first day of baseline. Ind showed a lower growth curve when compared to Con over the whole testing phase (F(1,15) = 6.3, p<0.05. * p<0.05. C) Food intake. Sub and dominants (Dom) mice under stress where hyperphagic when compared to baseline, Con and Ind mice (treatment, F(3,33) = 7.4, p<0.001; treatment x weeks F(9,99) = 3.8, p<0.001). In addition, Ind mice showed an overall lower level of kcal ingested when compared to controls. D) Visceral fat pads weight. Dom showed a smaller perigonadal (F(3,37 = 3.2, p<0.05), perirenal (F(3,37 = 3.2, p<0.05) and a trend for lower retroperitoneal (F(3,37 = 1.7, p = 0.1) pad weight than Con. * p<0.05, §p<0.07 vs. Con. E) Cumulative weight of visceral fat mass. Dom showed a reduction of visceral fat when compared to Con (F(3,37) = 2.3, p<0.1). * p<0.05 vs. Con.

In agreement with our previous report [26], the growing curves of Dom and Sub mice (Figure 3A) started to diverge soon after the beginning of stress procedure with Dom gaining less weight and Sub gaining more weight than control (Con) mice. The growing curve of both Dom and Sub was reduced in the week preceding the stress procedure onset and this might be attributed to individual housing [34 and see below]. Importantly, stress-induced hyperphagia emerged with both Dom and Sub mice that significantly increased the kcal ingested when compared to baseline (Figure 3C). As a result, both Dom and Sub ingested more kcal than Con and Ind mice during the stress phase (Figure 3C).

We dissected and weighted major visceral fat pads to determine the metabolic consequences of chronic stress and associated hyperphagia. Results proved that Dom but not Sub showed a marked decrease in the weight of perigonadal and perirenal fat pads while only a trend emerged for a lower retroperitoneal fat pad (Figure 3D). The mesenteric and the mediastinic fat pads remained unaffected. Overall Dom showed a lower content of visceral fat than Con (Figure 3E).

**Figure 4:**
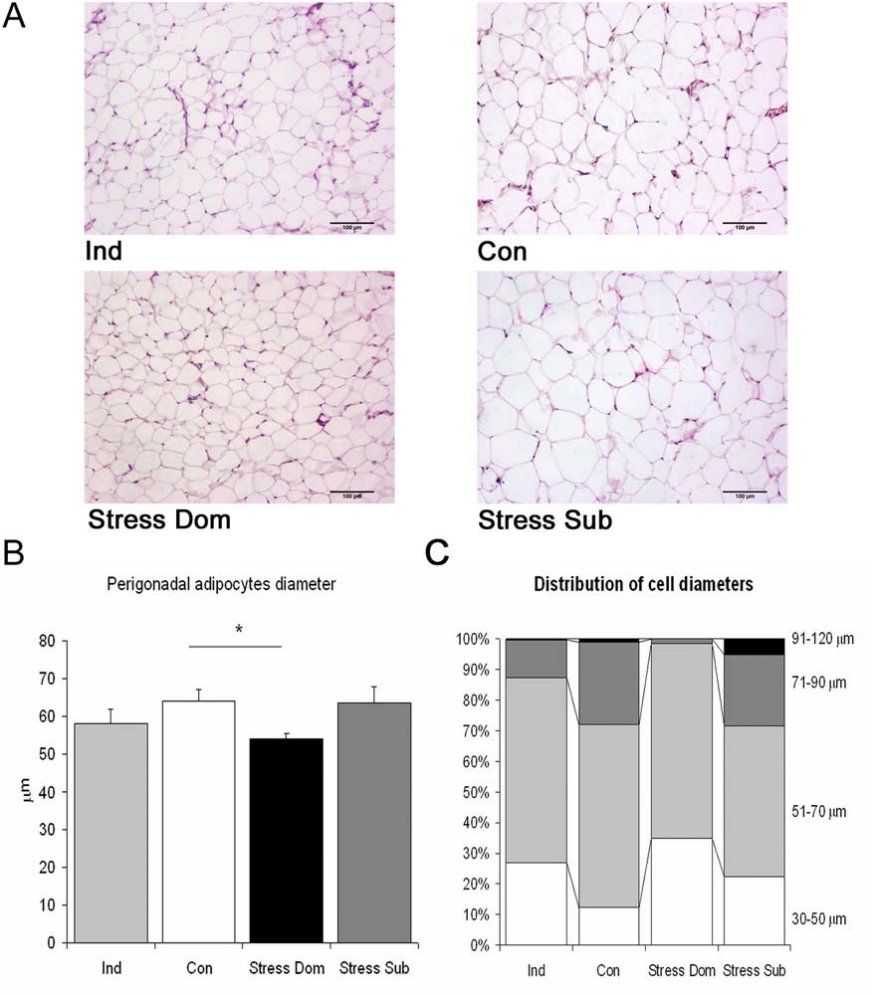
Effect of chronic stress on the histology of the perigonadal adipose tissue. A) Representative sections of perigonadal adipose tissue from individually housed (Ind), Control (Con), subordinate (Sub) and dominant (Dom) mice. B) Dom mice showed a significant smaller mean adipocytes diameter when compared to Con (U_10,10_ = 17, p<0.016), while all other groups remained unaffected. C) Categorized distribution of individual adipocytes diameters (see text for statistical details).

**Figure 5:**
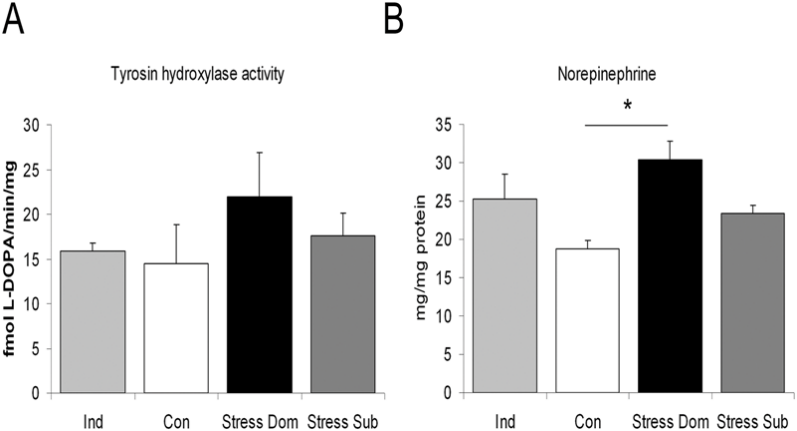
Sympathetic system related parameters in mice adipose tissue. A) Perigonadal adipose tissue tyrosine hydroxylase (TH) enzymatic activity assay revealed a small but not significant increase in the dominant (Dom) mice. B) Dom mice showed a higher perigonadal norepinephrine (NE) concentration than Controls (Con) (F(3,21) = 6.0, p<0.01). *p<0.05.

At the cellular level, Dom showed lower mean perigonadal adipocytes diameter when compared to both Sub and Con (Figure 4A,B). Furthermore, a quantitative analysis of individual adipocytes demonstrated that in Dom larger adipocytes (i.e. larger than 71 µm) were almost completely absent while they represented 20–30% of the adipocytes population in the other groups (a significant increase in 30–50 µm and a decrease in 71–90 µm sized adipocytes was observed, U_10,10_ = 15, p<0.0001 and U_10,10_ = 16, p<0.010 when compared to Con. Figure 4C). Furthermore, although the effect is quantitatively small, Sub showed an increase (from 0.5 to 1% in all groups to 5% in Sub) in very large adipocytes (i.e. larger than 91 µm. Figure 4C). This analysis revealed that dominant mice under chronic stress showed a clear adipocytes remodeling thus suggesting that the reduction in body weight may be due to sympathetic-driven lipolysis leading to overall reduction of adipocytes size and adipose tissue weight. To shed light on this hypothesis, we determined the enzymatic activity of tyrosine hydroxylase (TH), the rate-limiting enzyme in the biosynthesis of catecholamines, as well as norepinephrine (NE) concentration in perigonadal fat pads. Dom showed high NE concentration and a slight but not significant increase in TH activity while Sub showed no change in the same parameters (Figure 5). Furthermore negative correlations were found between final body weight gain and TH activity (r = −0.48, p<0.05) and NE concentration (r = −0.45, p = 0.05) as well as between NE concentration and perigonadal fat pad weight (r = −0.45, p = 0.05).

Overall, data from the present experiment proved that despite similar stress-induced hyperphagia Dom and Sub showed opposite metabolic consequences, i.e. Dom showed negative energy balance associated with increased sympathetic tone and locomotor activity which apparently were able to counteract hyperphagia, while Sub showed positive energy balance driven by hyperphagia and lower activity and being, thus, at risk for weight gain and obesity.

### Metabolic consequences of chronic individual housing

Ind mice showed a clear inhibition of weight gain when compared to Con under standard diet (Figure 3B). In addition when comparing the growing curve after the first seven days of individual housing (Figure 3A) (in analogy with Dom and Sub under chronic psychosocial stress), Ind mice only differed from Sub (lower weight gain) but not from Dom or Con. Ind mice ingested less kcal than Con mice for the duration of the whole experimental phase with values reaching significance in the last week (Figure 3C).

The weight of adipose tissue fat pads was generally reduced in Ind mice when compared to Con, though this effect was significant only for the perigonadal pad, while a trend emerged for the perirenal pad and no overall reduction of visceral fat pad was observed (Figure 3D and E). It must be noted, however, that in Ind mice neither changes in perigonadal adipocytes diameter nor any major change in the frequency of differentially sized adipocytes was noticed (Figure 4). Similarly, no change in TH activity or NE concentration in perigonadal fat pad was detected (Figure 5).

Therefore, in mice fed a standard diet, the effect of individual housing on weight gain were similar to those observed in mice that were maintaining dominance under chronic psychosocial stress. However, at variance with Dom, Ind mice showed a reduction in food intake, which seems to be largely responsible for the metabolic effects observed in absence of a hyperactivity of sympathetic-markers such as TH enzymatic activity and NE concentration.

Finally, in agreement with our previous report [34], Ind mice showed increased basal blood corticosterone concentration (Figure 2).

### High fat diet exposure

The observed status-dependent (Dom vs. Sub) and stress model-dependent (psychosocial stress vs. individual housing) metabolic consequences of stress suggest a possible differential vulnerability of Dom, Sub and Ind mice to diet-induced obesity (DIO) [28], [31]. To test this hypothesis, mice were challenged with a HFD that provides 45% kcal from fat and 5.2 kcal per gram (compared to the 6.5% and 3.9 values respectively of the standard chow) beginning on the first day of stress procedure or after 7 days of baseline (for Con and Ind). Based on the data obtained under standard diet conditions, we predicted that Dom and Ind should be less vulnerable, and Sub more vulnerable, to HFD-induced obesity when compared to Con.

**Figure 6:**
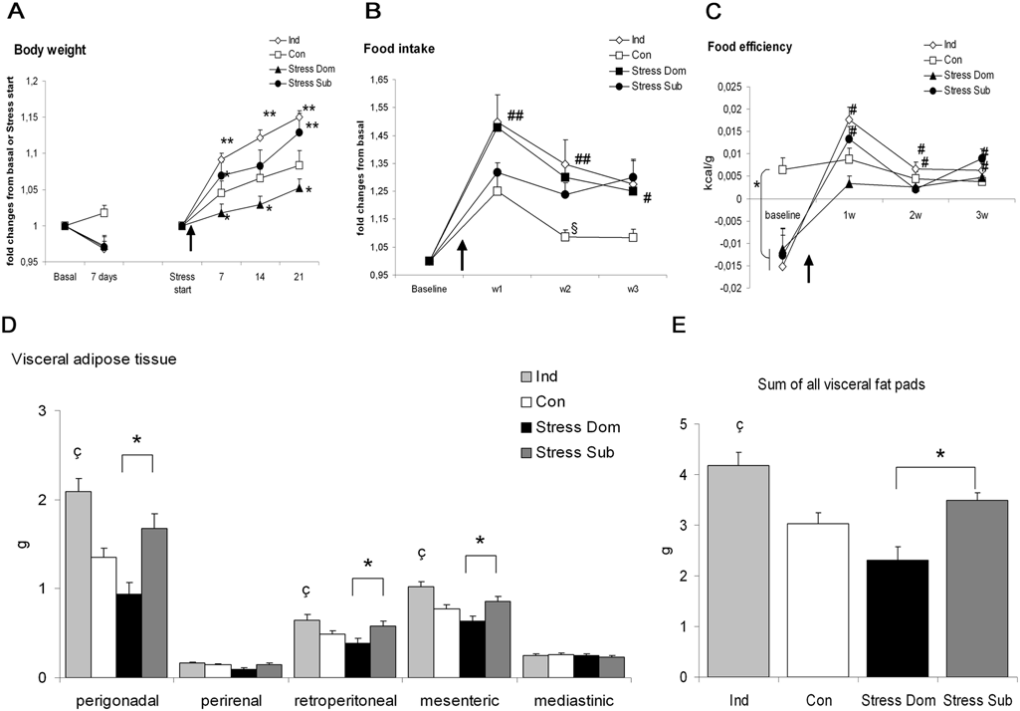
Vulnerability to high fat diet-induced obesity. A) Body weight changes in the baseline and in stress phase. At baseline, when mice were fed standard diet, all experimental groups showed a decrease in body weight, while controls (Con) showed a slight increase (F(3,23) = 3.2, p<0.05). In the stress phase subordinates (Sub) and individually housed (Ind) mice were more, and dominant (Dom) were less, vulnerable to weight gain than Con (F(3,23) = 5.3, p<0.01). In the graph only statistical comparison with Con are shown. In addition, both Sub and Ind mice differed from Dom (p<0.001) and Sub differed from Ind on day 14 only (p<0.05). B) Food intake. When animals were fed a high fat diet they showed a marked increase in kcal ingested. However a clear difference emerged between experimental groups (F(6,32) = 2.9, p<0.05) with Dom and Ind showing sustained hyperphagia when compared to Con along the entire experiment. Sub were hyperphagic only in the third week while showing a trend in the second week of the stress phase. Finally Sub also differed from Ind and Dom in the first week of the stress phase (p<0.01). C) Food efficiency analysis revealed that while Con were able to maintain a balance trough the changing dietary environment, Sub and Ind but not Dom significantly increased food efficiency with HFD (F(9,69) = 5.1, p<0.0001). D) Visceral fat pad weight. Dom showed an overall lower amount of perigonadal (F(3,23) = 9.2, p<0.001), perirenal (F(3,23) = 2.5, p<0.08), retroperitoneal (F(3,23) = 3.7, p<0.05) and mesenteric (F(3,23) = 7.2, p<0.005) but not mediastinic fat pad weight when compared to Sub. Ind showed a robust increase in perigonadal, retroperitoneal and mesenteric adipose fat pads which was significant versus Con and Dom but not versus Sub. E) Cumulative weight of visceral fat mass. Dom showed lower overall visceral adipose tissue than Sub. On the contrary Ind differed from Con and Dom but not from Sub (F(3,23) = 8.4, p<0.001). * p<0.05 and **p<0.01 vs. Controls, § p<0.07 vs. Controls, ç p<0.01 vs. Con and Dom. #p<0.05 and ## p<0.01 vs. Basal level for each group. Arrows describe the change from standard to high fat diet.

Indeed, results proved that Sub were more vulnerable and Dom more resistant to DIO than Con (Figure 6). Interestingly, this occurred despite Dom showing a 3 weeks-long hyperphagia while Sub being hyperphagic only in the last 2 weeks (Sub clearly ingested more kcal when compared to baseline throughout the 3 weeks period. Figure 6B). Contrary to our prediction, individual housing also determined an increased vulnerability to DIO. Indeed, Ind showed increased weight gain, hyperphagia and food efficiency when compared to Con (Figure 6). Therefore, despite Dom and Ind showing similar hyperphagia, the metabolic cost of dominance (as described in the previous section), was able to restrain food efficiency and avoid HFD-induced weight gain.

HFD resulted in a massive overall increase in adipose tissue weight when compared with mice fed a standard diet (see Figure 3E and Figure 6E). In particular, Dom showed lower perigonadal, retroperitoneal and mesenteric fat mass weight as well as overall visceral adipose tissue when compared with Sub. However, while a trend for Dom showing lower and for Sub showing higher fat mass than Con emerged, such effects did not reach statistical significance. In Ind mice, the adipose tissue was greatly enlarged, with perigonadal, retroperitoneal and mesenteric fat pads showing a greater increase than Con (Figure 6D), which also resulted in an overall increase in visceral fat mass (Figure 6E). Finally, Dom but not Sub showed lower adipose fat mass weight when compared with Ind (Figure 6E).

Overall, the data of Dom and Sub mice largely agreed with the prediction that Sub would have been more, and Dom less, vulnerable to HFD-induced obesity when compared to Con. In particular, HFD exposure increased the difference in adiposity between Dom and Sub, with Sub also showing slightly greater adipose mass than Con.

Data also proved that Ind mice were remarkably vulnerable to HFD-induced obesity and that exposure to hypercaloric and highly palatable diet was able to reverse the effects observed under standard diet, i.e. lower food intake and weight loss. The more likely explanation is that individual housing determined an increased hedonic response to high fat food and that: 1) the compensatory inhibition of initial hyperphagia (observed in controls) is disrupted in Ind mice (mechanism to be identified); 2) Ind mice are faced with a smaller metabolic cost than mice subjected to chronic psychosocial stress.

## Discussion

Social and psychological factors [36], [37] interact with genetic predisposition [38] and dietary habit [39] to determine the current obesity pandemia, and a possible link between chronic social stress, hedonism and vulnerability to obesity has been suggested [7]. However, up to now few pre-clinical studies directly addressed the role played by psychosocial factors and provided validated experimental models for human stress-induced metabolic disorders, which are very common, for example, in several psychiatric conditions [1], [4], [8]–[12]. In the present study we provided a comprehensive characterization of the metabolic consequences of social status under chronic psychosocial stress and social deprivation in male mice. Overall, our findings showed that in mice fed standard diet: 1) psychosocial stress determined opposite effects on energy balance, with Dom showing a negative and Sub a positive effect; 2) individual housing determined a reduction in weight gain; 3) hyperphagia emerged for Dom and Sub and hypophagia for Ind; 4) Dom showed increased NE concentration in fat tissue, lower perigonadal fat pad weight and smaller adipocytes diameter than Con. On the contrary, under high fat diet, Sub and, surprisingly, Ind showed higher, while Dom lower, vulnerability to obesity than Con.

Given the remarkable difference among the different experimental groups, data will be first discussed separately and then a general perspective on social modulation of metabolic functions will be provided.

### Chronic psychosocial stress: subordinate mice show positive energy balance and increased vulnerability to diet-induced obesity

Subordination-induced weight gain is not a common observation in animal models of chronic social stress [19]–[24]. Indeed, we were the first to describe a subordination-induced weight gain in mice during the chronic psychosocial stress procedure [26], a finding that has now been replicated by other groups using similar preclinical animal models of social stress [27]–[30], [40]. This discrepancy in subordination-stress induced positive o negative weight changes does not have a clear explanation at the moment. However, when assessing the literature there are a number of factors that should be taken into account. Firstly, changes in body weight are often the sole metabolic parameter presented and it is difficult to interpret a decrease in body weight without a control for feeding, locomotion or energy expenditure. Secondly, it appears that the species and the strain investigated may play a role, since most of the data showing weight loss have been obtained with subordinate rats or tree shrews and only a few with mice [41]–[43]. Among the mouse studies none was performed with the CD-1 strain. Thus the results presented here raise the possibility of a strain-associated vulnerability to stress-induced weight gain. However, we recently obtained very similar subordination-induced metabolic effects on inbred strains of mice (Bartolomucci et al., unpublished observations) thus suggesting that positive vs negative changes in energy balance is probably primarily dependent on the model of stress used rather than on the strain. Thirdly, the experimental animals are generally faced with an unstable aversive environment with the experimental procedure often requiring a brief daily move into the dominant home cage with individual housing for the rest of the day [21], [22]. In other studies the subordinate is moved daily, or every second day, into different dominant cages [20], [41]–[43]. Thus other models of social stress may determine a mixed subordination/individual housing/instability effect with major inhibitory effects on feeding (see also below). Finally, when data on feeding have been collected, weight loss in subordinate rats was associated with a reduction in feeding [24], [44], [45], while post-stress hyperphagia and weight gain has been reported for subordinate rats in the visible burrow system [46].

In our experimental setup, body weight changes were associated with hyperphagia in Sub mice, similarly to what has been previously reported [27]–[29], [40]. In addition, we have previously shown similar food consumption in Dom and Sub under stress [26]. In agreement with our previous report [47] Sub also showed a reduction in locomotor activity during stress exposure, which is reminiscent of the psychomotor impairments and reduced willingness to engage in daily activities observed in depressed patient [15], [44]. Therefore, results from the present and previous studies, prove that positive energy balance in Sub is associated with increased feeding and lower activity. Surprisingly, increased body weight gain in Sub did not translate into higher fat pad weight. This finding is in agreement with our previous report [26] and suggests that alterations in subcutaneous adipose tissue, water content or lean mass might be responsible for the increased weight gain, but rules out a primary role for visceral adipose tissue in explaining increased body weight. This lack of effect on visceral adiposity is also surprising because Sub showed increased circulating corticosterone which is know to be associated with increased visceral adiposity [1], [4], [5]. However, it is of interest to note that Sub mice showed an increased number (although not significant) of very large sized adipocytes (i.e. larger than 91 µm in diameter) in the perigonadal pad, which can be considered as an incipient hypertrophic obesity [48], [49] possibly leading to increased vulnerability to cell death [50]. Finally, in our model Sub show a similar up-regulation of HPA axis as well as tachycardia than Dom [reviewed in 14], [16; present data]. Accordingly, the adipose tissue is probably exposed to opposing stimuli that may result in the lack of a net effect on adipose fat pad weight.

On the contrary, when subordinate mice were fed HFD, the result was an increase in weight gain in the late phase of the stress procedure and a consistent increase in adiposity. HFD determined a generalized hyperphagia in the second and third week of stress likely explaining the delayed effect of HFD on weight gain. Therefore, subordination under chronic stress may represent a vulnerability factor for diet-induced obesity.

Overall, our data indicate that subordinate male mice under chronic stress represent a valid model of stress-induced depression-related disorders [15], [16]. As well, our data also validate the conclusion that chronic psychosocial stress represents a model of stress induced weight gain and vulnerability to obesity. These data find a parallel also in primate and human literature. In a recent study with rhesus macaque, Wilson and coworkers [51] showed that subordinates gained more weight and dominants gained less weight than controls under both low and high fat dietary regimen and that subordinates were hyperphagic. Finally, in the human literature it has been repeatedly reported that psychosocial and socio-economic challenges such as low income, low education and divorce have been associated with perturbed cortisol secretion, over-eating, metabolic syndrome and type 2 diabetes [1], [52]–[54].

### Chronic psychosocial stress: dominant mice show negative energy balance, sustained sympathetic activity in the visceral adipose tissue and resistance to diet-induced obesity

In the present experimental context, Dom mice showed a negative energy balance associated with hyperphagia. Evidence for a high cost of dominance in our experimental protocol comes from both behavioral and biochemical results. Indeed, Dom showed a marked behavioral hyperactivity in the stress phase both in the light and in the dark period. Previous studies also demonstrated that Dom showed a strong increase of sympathetic function as indicated by tachycardia, hyperthermia, and increased energy expenditure [26], [28] as well as hyperphagia [28]. In addition, Sakai and co-workers [23], [24], reported that dominant rats housed in the visible burrow system model of chronic stress showed a slight decrease in body weight and a reduction in adiposity, which was associated with higher feeding than subordinate rats [46].

No study had previously investigated sympathetic system related parameters in the adipose tissue of mice under chronic stress. The white adipose tissue (WAT) is innervated by the sympathetic nervous system and a direct role for WAT sympathetic noradrenergic nerves in lipid mobilization has been demonstrated [46], [55]–[57]. Here we showed that perigonadal WAT NE concentration and, to a lesser extent, also the activity of the rate limiting catecholamine-synthesizing enzyme TH [58], were increased in Dom. Increased sympathetic markers in the adipose tissue have previously been associated with catabolic processes and weight loss [48], [56], [59], [60]. In agreement with a direct role of NE in regulating the adipose organ, here we demonstrated that Dom showed a decrease in perigonadal, perirenal and retroperitoneal, but not in mesenteric and mediastinic fat pads, thus supporting a strong regional difference in sympathetic nervous system activity on adipose tissue [56], [61]. In addition Dom also showed lower mean adipocytes diameter, and a classification of perigonadal adipocytes based on their diameter revealed that Dom showed an apparent disappearance of large adipocytes (greater than 71 µm). These findings, in addition to increased NE concentration in the same fat pad, suggests that a sympathetic mediated lipolysis is the primary cause of the reduction of fat mass in dominant mice under chronic stress. In this respect, it is of interest to note that NE was negatively correlated with final body weight gain and with perigonadal fat mass. Finally, the sustained metabolic cost associated with maintaining dominance under stressful conditions also translated in a resistance to HFD-induced obesity. Dom showed lower weight gain, and lower adipose weight associated with remarkable hyperphagia, thus supporting the conclusion that sustained behavioral and sympathetic activity might limit diet-induced obesity.

In conclusion, present data further strengthen the conclusion that maintaining dominance in stressful conditions is strongly associated with a physiological cost [16], [62]–[64]. Central pathways determining sustained sympathetic stimulation have not been determined in the present study but increased CRH/AVP signaling and hyperactivity of the melanocortin system [65], [66] is fully compatible with both high aggressive level/dominance and negative energy balance leading to lipolysis [67].

### Individual housing: opposite feeding response and metabolic consequences with standard or high-fat diet

Individual housing is often considered a model of social stress in rodents because of the factual deprivation of social contacts [34], [68]–[70]. Previous reports from our [34] and other groups [71]–[73] proved that individual housing is associated with a negative energy balance with animals loosing weight or maintaining a lower weight gain than group housed siblings. In this study, we provided a detailed investigation of metabolic functions associated with individual housing and proved that: 1) in mice fed a standard diet, isolation is associated with a reduction in food intake and a decrease in perigonadal fat pad. Reduced feeding, lack of social facilitation of feeding [74], and unbalanced thermoregulatory functions associated with lack of social contact [75], [76] are the likely factors responsible for the decrease in body weight; 2) Ind mice fed HFD responded with sustained hyperphagia and increased vulnerability to diet-induced obesity resulting in 16% weight gain and a massive increase in adipose fat pad weight.

Therefore, it is apparent that reduced food intake under standard feeding regimen can be due to lower social facilitation to initiate the feeding [74] rather than to an overall negative motivation to feed [77]. Indeed, when mice were provided with a highly palatable diet they responded with conspicuous overfeeding as previously observed with cafeteria diet [78]. There are very few investigations on the metabolic consequences of individual housing [34], [71]–[73], [78]. In a recent important study Nonogaki and coworkers [31] reported an impressive strain difference in the vulnerability to weight gain induced by social isolation. Indeed, the authors proved that: 1) individual housing was associated with increased weight gain and overfeeding in the KK strain and in KK mice carrying the ectopic overexpression of agouti (KKA^y^); 2) the C57BL6/J strain showed no effect of individual housing; 3) individually housed diabetic db/db mice, carrying a mutated leptin receptor gene, showed lower body weight and hypophagia when compared with group housed db/db. Our model using an outbred strain may recapitulate the variability described by Nonogaki and coworkers and suggests that at the “population” level, male mice are vulnerable to obesity only when faced with HFD. This model also complements recent evidence [79] showing that epigenetic mechanisms might be more important than genomic differences in explaining a large proportion of individual vulnerability to obesity.

### An overall view: social stress affects metabolic function in male mice

**Figure 7:**
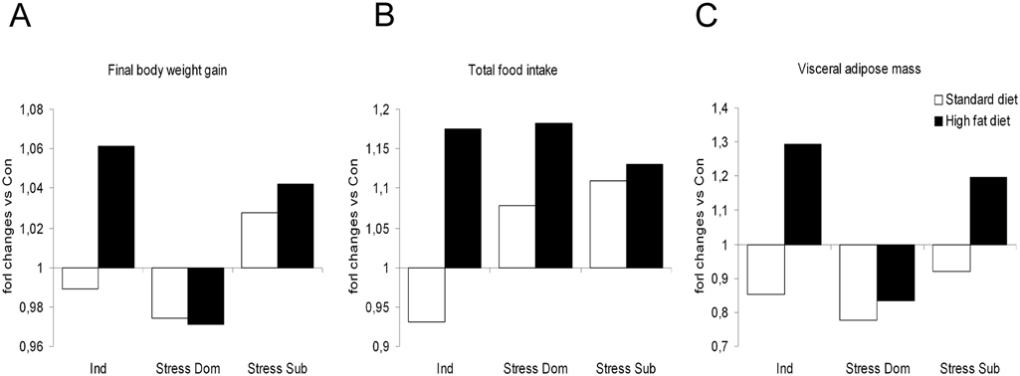
Overview of the metabolic effects induced by chronic psychosocial stress and individual housing. The graph shows variation (versus the mean value of the control group-housed mice) for body weight changes, food intake and total visceral adipose fat mass weight, under standard or high fat diet. Individual housing (Ind) determined negative or positive energy balance depending on the diet being standard or high fat diet respectively. Dominance (Dom) determined a similar negative energy balance with both standard and high fat diet. Subordination (Sub) determined similar positive energy balance with both diets. However, body weight gain and feeding were similarly affected under standard and high fat diets while visceral fat pad mass increased with high fat diet only.

In the present study we directly compared different models of social stress and described major metabolic alterations associated with dominance, subordination and individual housing (Figure 7). Overall data proved that: 1) subordinate mice under chronic stress showed increased weight gain without increased visceral adiposity under standard diet and increased vulnerability to obesity with HFD; 2) dominant mice under chronic stress showed lower weight gain and reduced adipose tissue independently from the feeding regimen; 3) individual housing resulted in lower weight gain and adiposity with standard chow and massive vulnerability to obesity with HFD; 4) group housed sibling mice (our control group) showed large fat mass under standard diet but lower vulnerability to HFD-induced obesity when compared to Sub and Ind. The latter result is important because it demonstrates that although CD-1 are among the heavier laboratory strain of mice, psychosocial stress exposure is sufficient to increase vulnerability to HFD-induced obesity.

Our data also provide direct confirmation to a model linking allostatic load to metabolic disorders recently proposed by Van Dijk and Buwalda [32]. This model states that metabolic syndrome and obesity can develop in presence of a high fat regimen only when an environmental threat prevents active coping (fight/flight) but permits only a passive strategy. Indeed, in our experimental model both Dom and Sub are faced with a threatening situation, and show similar overactive HPA axis and cardiac hyperactivity as well as hyperphagia, while: a) dominants responded with an active coping style associated with sympathetic overactivity in metabolic tissues that limited the development of obesity despite overfeeding; b) subordinates instead responded with a passive helplessness strategy and, particularly when faced with a high fat diet, developed weight gain and obesity. Indirect confirmation comes from the profile of Ind mice (considered a model of mild depression [34], [69]–[72]) which showed lower feeding and body weight gain in the absence of any sympathetic hyperactivation when fed chow diet while becoming hyperphagic and obese in the presence of HFD.

Although the molecular and endocrine mechanisms responsible for metabolic disorders are currently unknown, present data clarify the role of social factors in modulating the individual vulnerability to weight gain and offer an important experimental tool for the investigation of the mechanisms linking stress and psychological disorders to metabolic dysfunctions.

## Methods

### Overview of the experimental procedure

Adult male mice were individually housed (Ind), group housed in groups of 3 siblings (here considered as the control group, (Con) [33], [80]) or were submitted to chronic psychosocial stress [26], [32] and identified as dominant (Dom) or subordinates (Sub) by behavioral observations. The experimental phase consisted in a baseline phase and in a stress phase (were animals were fed standard or high fat diet). Body weight, food intake and locomotor activity (in Sub and Dom only) were determined (see below). Subsequently on day 20 mice were behaviorally tested in the modified open-field test and the following morning sacrificed. After termination, adipose fat pad weight, tyrosine hydroxylase (TH) activity and norepinephrine (NE) concentration in the perigonadal fat pad along with histological determination of adipocytes diameter were obtained. Finally, plasma level of corticosterone was determined.

### Animals

Subjects were adult male Swiss CD-1 mice from an outbreed stock originally obtained from Charles River Italia (Calco, Italy). Mice were born and reared in a colony room at the University of Parma at 22±2°C in a 12-hr light–dark cycle (lights on 0700-1900). After weaning (25–28 days of age) they were housed in same-sex- groups of siblings (4–7 per cage) in Plexiglas cages (38×20×18 cm) with wood shaving bedding changed weekly. All animal experimentation was conducted in accordance with the European Communities Council Directive of 24 November 1986 (86/EEC) and approved by the Ethical committees of the University of Parma and the Italian Institute of Health.

### Chronic Psychosocial stress

The procedure has been originally described by Bartolomucci et al. [33] and was used here with minor changes to adapt to specific requirement of metabolic studies. Three-months old male mice to be used as residents or intruders, were individually housed in Plexiglas cages (38×20×18 cm) for a 7 days baseline phase. To allow recording of baseline individual locomotor activity, after day 1 a wire-mesh partition bisecting the cage longitudinally was introduced. This restricts the access to only half the cage to mimic the conditions of the stress phase (detailed below). On day 6 of the baseline phase, the wire-mesh partition was removed to give the animal access to the entire cage thus allowing re-establishing of individual territory in the whole cage. Baseline, body weight and food intake were monitored at the beginning and the end of the 7 days. On day 7 the 21 days stress phase begun and each resident mouse received an unfamiliar same-sex weight-matched intruder mouse and the two animals were allowed to freely interact for 10 minutes. In order to prevent injuries, the social interaction was interrupted if fighting escalated (when the dominant persistently bit the opponent). After the interaction, the two animals were separated by means of a wire-mesh partition, which allowed continuous sensory contact but no physical interaction. The partition bisected the cage longitudinally in two symmetrical compartments. Between 10:00 and 12:00 hours the partition was removed daily for 10 min. Throughout the stress phase body weight was monitored weekly, food intake was monitored daily and locomotor activity was monitored continuously except during the aggressive interaction. Throughout the study food and water were available ad libitum to all experimental mice.

During the social interaction offensive behaviors of the animals were manually recorded and mice social status was determined as follows: the chasing and biting animal was defined as ‘Dominant’, while the mouse displaying upright posture flight behavior and squeaking vocalization was the ‘Subordinate’. The numbers of attack bouts performed by each animal were quantified during the first four days than again at day 10 and 20 by direct observation. When the fight has to be interrupted before the 10 min, the number of attacks was computed proportionally. Four behavioral categories were distinguished within the stress group: (i) resident dominant, (ii) resident subordinate (RS), (iii) intruder dominant, (iv) intruder subordinate (InS). Previous studies showed minor differences in the metabolic functions of RS and InS mice and no difference between the two dominant categories [16]. Although RS had the largest effects in terms of body weight gain and adiposity [26], there was no statistical difference between the two groups (which on the contrary largely differ in immune function [16]). In addition, the present study confirms no significant difference between RS and InS (data not shown). Therefore RS and InS were pooled in the group “Sub” and the two dominant categories in the group “Dom”.

Age-matched mice, housed in groups of 3 siblings, were included as the non-stressed control group (Con). This choice was based on previous observations showing no metabolic, immune-endocrine and behavioral evidence of stress activation or anxiety in group-housed siblings (see [33], [34], [80] for details). Within each control group, the hierarchical status of the animals was determined according to [33], and then the dominant and one of the two subordinate mice (randomly chosen) were used for experimental measurements. Data from this experiment confirmed absence of status-associated effects between dominant and subordinate mice in groups of siblings (data not shown).

### Individual housing

Three-months old male mice were individually housed in Plexiglas cages (38×20×18 cm). Body weight was monitored weekly and food intake daily. Controls were the same age-matched mice housed in groups of 3 siblings described above.

### Home cage locomotor activity

The assessment of individual daily activity was carried out by means of an automated system that use small passive infrared sensors positioned on the top of each cage (TechnoSmart, Rome, Italy). To avoid interference between the movement of a resident and an intruder mouse in the same cage the two individual sensors were separated by a Plexiglas partition which completely blocks infrared waves. The system was set-up prior to the beginning of the experimental procedure to verify absence of false signals across adjacent sensors (data not shown). Locomotor activity was continuously monitored throughout the whole experiment including 4 days of baseline phase and 20 days of stress phase. Recording was interrupted only during the daily agonistic interaction.

### Modified open field test

The test was performed between 16:00 and 19:00 of day 20, in agreement with Berton et al [81] with minor changes. Each experimental mouse was introduced into a squared open field (54×54 cm) for two consecutive sessions of 2.5 min. During the first session (T1, “target cage empty”) the open field contained an empty wire mesh target cage (10 cm diameter) located at one end of the field. During the second session (T2, “intruder mouse present”), the conditions were identical except that a social target animal (a same age unfamiliar CD-1 male mouse) had been introduced into the cage. Between the 2 sessions, the experimental mouse was removed from the arena, and was placed back into its home cage for approximately one minute. Mouse behavior was scored with Ethovision (Noldus, the Netherlands). Within the arena the following area were identified and time, frequency and latency determined: “target zone” (an 8 cm wide corridor surrounding the target cage); the “far corners” of the open field opposite to the location of the cage; the four corners. All CD-1 mice independently from the experimental treatment spent around 70–80% of the time in the target zone (data not shown) with no group difference in avoidance/approach time ratio spent in the target area between T1 and T2. On the contrary, using C57BL6/J mice the procedure determined similar response as described by Berton et al [81] (Bartolomucci et al., unpublished). This finding highlights a major strain difference (C57BL6/J vs. CD-1) in the behavioral response to an object located within the arena. Thus, a procedural modification is needed to investigate the behavior of CD-1 mice in this behavioral test. Because of this limitation data from this test are not presented. Nevertheless, the test is discussed here because previous data from our group revealed that corticosterone level in Ind mice are particularly sensitive to the acute exposure to an open field [33].

### Diet

Mice were fed a standard (6.55% kcal from fat and 3.9 kcal/g; 4RF21, Mucedola, Italy) or a custom pelletted high fat diet (45% kcal from fat and 5.2 kcal/g manufactured by Mucedola) modifying the formula of the standard diet 4RF21.

### Adipose organ parameters

Adipose fat pads (perigonadal, perirenal, retroperitoneal, mesenteric and mediastinic [82]) were manually dissected and weighted. Perigonadal pads were split in two parts and one half was snap frozen in liquid nitrogen and stored at −80°C for later measurement of sympathetic related parameters (see below). The second half was immerged in a ice-cold solution of 4% paraformaldeyd, stored at −4°C overnight and processed for histological analyses (see below).

### Norepinephrine concentration and tyrosine hydroxylase activity

TH activity in adipose tissue was analyzed by the method of Naoi et al [83]. Biopsies were homogenized and incubated at 37°C for 10 minutes with 140 µmol/L L-tyrosine in 880 µL of sodium acetate-acetic acid buffer (100 mmol/L, pH = 6.0) containing 1.4 mmol/L (6R)-5,6,7,8-tetrahydrobiopterin, 10 µg of catalase, and 0.7 mmol/L 4-bromo-3-hydroxybenzyloxyamine (NSD1055, an inhibitor of aromatic L-amino acid decarboxylase). The incubation was stopped by the addition of 0.1 mmol/L perchloric acid containing 0.4 mmol/L sodium metabisulphite and 0.1 mmol/L disodium EDTA. After vortexing, the sample was allowed to stand in an ice bath for 10 minutes and then centrifuged at 1000 g for 10 minutes. The supernatant was injected in a HPLC-ECD system for L-3,4-dihydroxyphenylalanine (L-DOPA) analysis. TH activity was calculated as the amount of L-DOPA generated from L-tyrosine per minute per milligram of tissue. NE was measured by HPLC using electrochemical detection, as previously described [84].

### Histological analysis

Specimens of perigonadal adipose tissue from different mice were carefully removed, weighted and immersed in 4% paraformaldehyde, dehydrated in ethanol, transitioned in xylene, and embedded in paraffin. Five-micrometer-thick sections cut with a cryostat were stained with hematoxylin and eosin. Optical microscopy images (Nikon Microscope Eclipse 80i) were digitally captured with NIS-Elements imaging software F 2.20, and the diameter of 200 adipocytes for each mouse was measured with ImageJ software (Image Processing and Analysis in Java).

### Analysis of Corticosterone

Trunk blood was collected in heparinized tubes, centrifuged at 4,000 RPM for 10 min and plasma was frozen at −20°C for later analysis. Level of circulating corticosterone was measured in duplicate with a commercially available RIA kit (Diagnostic Systems Laboratories, Inc., USA) with a sensitivity of 0.06 ng/ml. The intraassay variability was 3.4%. To avoid the interassay variability, all samples were run in a single assay.

### Statistical analysis

Data were checked for agreement with parametric assumption and analyzed with ANOVA followed by Tukey's HSD post hoc or Mann-Whitney U test with the Bonferroni correction when appropriate. Correlations were performed with parametric Pearson test.
